# Mandibular Molar Uprighting Using Skeletal Anchorage: A Novel Approach

**DOI:** 10.3390/jcm11133565

**Published:** 2022-06-21

**Authors:** Luca Sbricoli, Sara Ricci, Andrea Cattozzo, Riccardo Favero, Eriberto Bressan, Stefano Sivolella

**Affiliations:** Department of Neurosciences, School of Dentistry, University of Padua, 35122 Padova, Italy; sararicci04@hotmail.it (S.R.); cattozzoandrea78@gmail.com (A.C.); rickyfavero@msn.com (R.F.); eriberto.bressan@unipd.it (E.B.); stefano.sivolella@unipd.it (S.S.)

**Keywords:** molar uprighting, orthodontic miniscrews, TADs, oral surgery, orthodontics

## Abstract

Background: The management of eruption anomalies affecting second molars, although quite uncommon, may represent a real challenge for the clinician. The aim of this study is to present a novel technique that combines the surgical and orthodontic approach in order to obtain the retrieval of impacted second molars through a complex distalizing movement and skeletal anchorage. Methods: Nineteen consecutive patients with impacted second molars were treated according to this technique, which involved extraction of the third molar followed by the placement of a distally positioned screw, and the subsequent use of a coil spring to connect the screw to an eyelet directly bonded on the second molar. In selected cases, it proved to be necessary to reposition the second molar through traditional orthodontics. All of the parameters were recorded: the time required for completing the treatment, the initial angle between the second molar and the adjacent tooth, and treatment related-complications. Results: Twenty impacted second molars were correctly repositioned. The mean initial angle of the second molar was 55.7° (SD 22.6°, min 13.3°, max 104.8°). The mean treatment time was 10 months. There were no major complications. Conclusions: Given that periodontal tissues were minimally affected, the temporary anchorage device was fully controlled, and there was no unwanted movement of adjacent teeth, the presented technique not only proved effective but also minimally invasive.

## 1. Introduction

The permanent teeth that are mostly affected by eruption problems are the mandibular and maxillary third molars, the maxillary canines, the lower premolars, the central incisors, and (more rarely) the mandibular second premolars [1,2]. Eruption problems affecting the second molars are quite rare, accounting in the literature for 0.03–0.4% of all impacted teeth [3]. Impaction of the second molar occurs much more frequently in the mandible than in the maxilla. This may be attributable to the different eruption pathway of the lower third molar [4]. This eruption problem tends to be monolateral. Varpio and Wellfelt found more cases on the right side; Cho et al. on the left [4,5]. Concerning sex prevalence variability, reports in the literature seem to be contradictory [4,5,6,7,8].

Andreasen identified three main causes of impaction of the lower second molar: ectopic positioning, obstacles in the eruption path (impacted third molars, supernumerary teeth, cysts or tumors) and failure of the eruption mechanism (ankylosis or laceration) [9]. Posterior crowding is considered the most common cause of impaction of mesially angled lower second molars [10].

Incorrect cemented banding on the mandibular or maxillary first molar after orthodontic sagittal expansion is also an important iatrogenic factor [11]. Since the eruption of the second molar must be guided by the roots of the first molar, an excessive amount of space could represent another cause of impaction [12]. Eruption problems, for example, may occur in those cases in which there is too much space between the two teeth after an orthodontic expansion of the maxillary arch. The second molar may also develop spontaneous eruption anomalies due to an excessive mesioversion of the tooth germ or due to the presence of a third molar characterized by an early crown development and position. Other issues may be related to premature first permanent molar extraction, molar ankylosis, odontogenic cysts or odontomas [8]. The hereditary factors that are potentially involved in the eruption anomalies affecting second molars include systemic conditions and dental abnormalities [13].

Second molar malpositioning is a relatively rare dental condition, and its clinical management has not yet been validated through a generally accepted strategy. In the past, authors proposed various approaches to the surgical-orthodontic retrieval of impacted mandibular second molars [14,15]. All of these approaches involved the use of a traction closed coil spring and only differed in the point of application of the traction force. The surgical retrieval of unerupted second molars could be anticipated by an operculectomy, according to Abate et al., to ease the spontaneous eruption of the second molar in 8.5% of the cases [16].

A great innovation in the orthodontic traction field came with the introduction of temporary anchorage devices (TADs). Lee et al. and Giancotti et al. described the use of miniscrews as an “absolute” skeletal anchor for uprighting impacted second molars [17,18]. The orthodontic uprighting of second molars can benefit from bone remodeling, which is consequential to the extraction of any third molar [19,20]. The placement of a submerged TAD distal to the third molar allows identification of an ideal traction point for distal movement. This technique enables avoidance of any soft tissue complication that is often associated with the use of such devices.

The aim of this retrospective case series is to present a novel technique, based on skeletal anchorage combined with the surgical extraction of the adjacent third molar, that allows uprighting of impacted second molars.

## 2. Materials and Methods

### 2.1. Patient Selection

Patients were screened between January 2016 and June 2021, and all patients that required surgical uprighting of impacted lower second molars were taken into consideration. Impaction was diagnosed on clinical grounds (failure of the permanent tooth to erupt in the arch within the physiological time frame) and radiographic evidence (orthopantomography; cone-beam tomography in those cases that demanded assessment of the relationship between the second and third molars and the inferior alveolar nerve).

### 2.2. Surgical and Orthodontic Procedures

**Figure 1:**
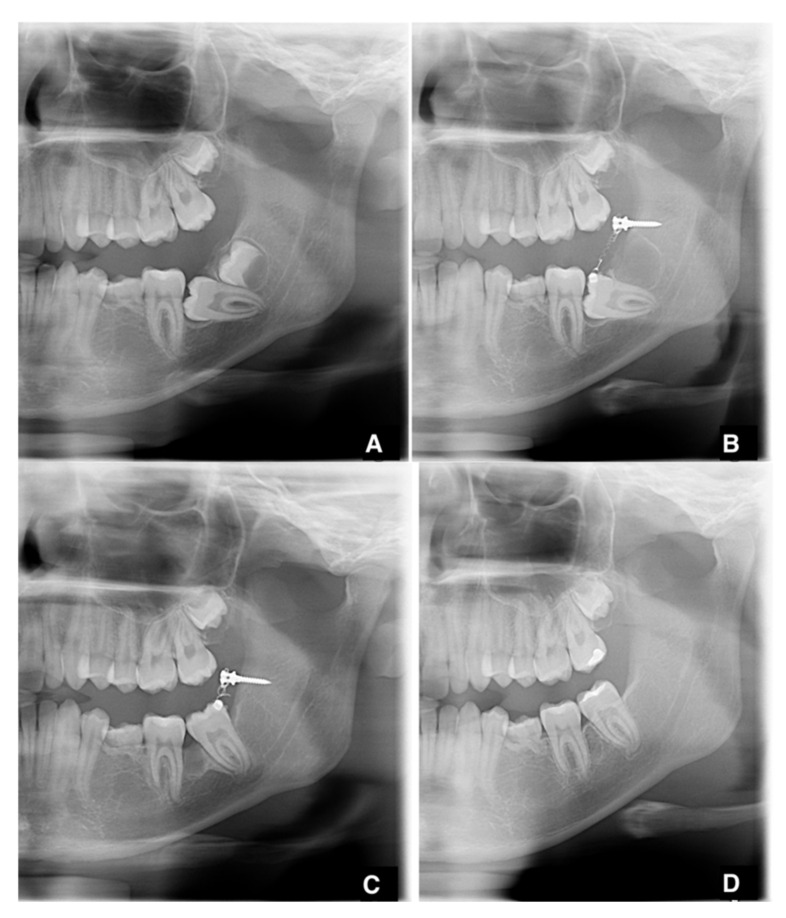
(**A**–**D**) The whole radiographic sequence of intervention (case 1).

**Figure 2:**
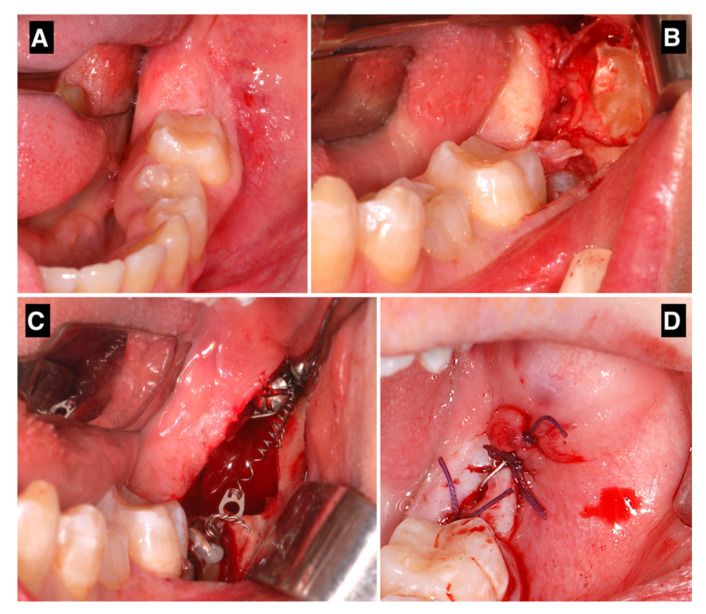
(**A–D**) The surgical procedure, from the initial condition to flap closure.

**Figure 3:**
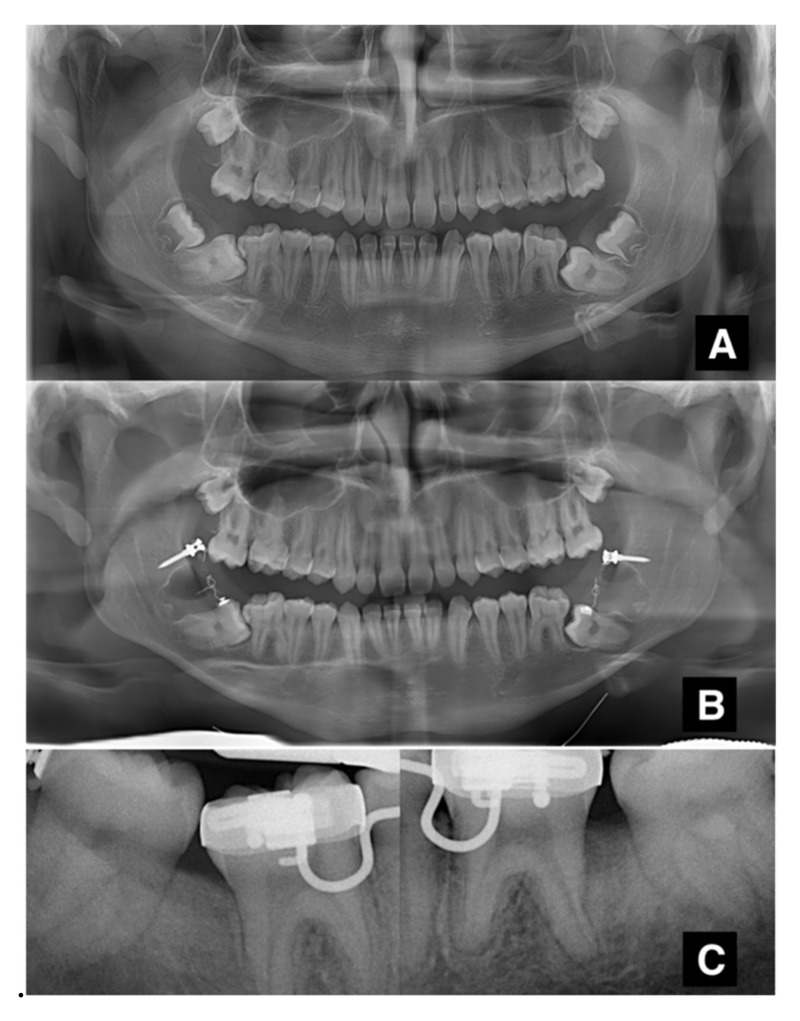
(**A**–**C**) Radiographic sequence of a rare bilateral case.

After performing an inferior alveolar nerve block anesthesia with mepivacaine without adrenaline, and local anesthesia with mepivacaine with adrenaline 1:100,000, a full-thickness, distally extending flap was elevated, and the ipsilateral third molar was extracted (Figure 1A). Simultaneously to the extraction, a sterile orthodontic screw was inserted posteriorly to the extraction site, while ensuring to avoid damaging the inferior alveolar nerve (Figure 1B). The screws were 2.0 mm in diameter, with thread lengths of 8–12 mm (the length of the screws was chosen according to the measurements taken on the available radiographical documentation) and made of 316 L extra-hard stainless steel for maximum strength. In order to perform a one-step insertion, we selected self-drilling and self-tapping screws, with grooves under their head for securing wires or elastic bands. The cruciform screw head was made of two cross-holes aligned with the head slots and a 4 mm capstan-style head to hold the wire away from the mucosa (Synthes, West Chester, PA, USA). When required, a limited ostectomy was performed to expose the crown of the second molar (Figure 1C), thus allowing a better movement of the tooth. An orthodontic button with an eyelet was bonded to the vestibular or occlusal aspect of the impacted second molar, and its position was chosen according to the direction of traction needed to upright the impacted tooth. A 200g NiTi closed coil spring was secured both to the miniscrew and the button. Before applying the suture, it was fundamental to ensure that the device would not interfere with the centric occlusion and the lateral mandibular movements. The flap was closed with single sutures, completely submerging the screw, the coil spring and, in most cases, the second molar (Figure 1A–D). Antibiotics (amoxicillin 875 mg and clavulanic acid 125 mg) were prescribed twice a day for seven days. An orthopantomography was taken after the surgical procedure to check the final position of the screw. Sutures were removed after seven days. Patients were instructed to pay very careful attention to their oral hygiene and to perform chlorhexidine 0.2% mouthwashes twice a day for a week. Patients were recalled every 3 weeks for orthodontic assessment, and a periapical X-ray was taken every six weeks, to assess the stability of the device and the position of the second molar. Patients were warned about the possibility of undergoing a second surgery to restore the traction whenever the device proved to be unstable (e.g., screw loosening, coil fracture, button detachment). When the second molar was moved in the desirable position, the uprighting device was removed by creating a small flap or using a diode laser, under local anesthesia. Multibracket orthodontic finalization was used in cases of sub-optimal final positioning. The whole radiographic sequence is shown below (Figure 2A–D). The same protocol was maintained for bilateral retained molars (Figure 3A–C).

### 2.3. Measurements

**Figure 4:**
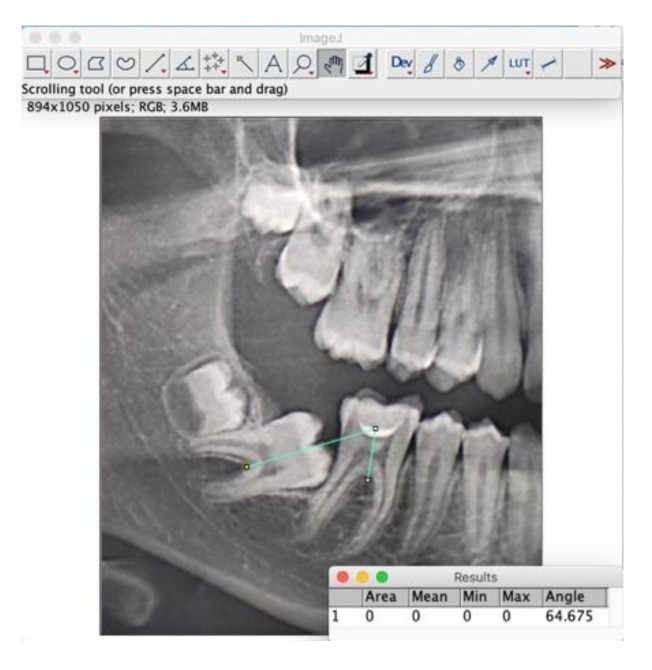
Radiographic measurement performed using ImageJ software.

Digital panoramic, cephalometric radiographs (Orthophos XG, Sirona Dental Systems Inc., Long Island, NY, USA) and study models were obtained at the baseline. A digital panoramic radiograph was obtained after the treatment was concluded. As reported by Sonis and Ackerman [20], panoramic images were magnified 200% to facilitate their analysis with diagnostic software. The angle of all second mandibular molars was assessed through the construction of a computer-generated intermolar angle. This angle is calculated between the first and second molars using the long axis of each tooth, based on a right-angle line bisecting the mesial-distal dimension of the crown at its widest point (Figure 4) (ImageJ software, National Institute of Health USA, http://imagej.nih.gov./ij accessed on 20 May 2022. The presence or absence of third molars was recorded.

## 3. Results

Nineteen patients with eruption problems involving the lower second molar (eight females and eleven males; mean age 18 years and 6 months at the baseline) were treated using the above-described technique. Table 1 shows the sample’s demographic details. The right second molar was involved in 11 cases, the left second molar in 7, and both in 1 (for a total of 20 impacted second molars). The third molar was absent in only two cases (10%). The mean initial angle of the second molar was 55.7° (SD 22.6°, min 13.3°, max 104.8°). No inferior alveolar nerve lesions or major surgical complications were reported. Minor complications (mucosal hypertrophy due to poor oral hygiene) occurred in three cases (15%). At the control visits and at the removal of the device, no detachments of the molar button and no loosening of the screw were observed, confirming the previous radiographic evaluations. The removal of the screw was always uneventful, and no fractures of the device occurred. The average duration of the orthodontic traction, from the miniscrew’s application to its removal, was 10 months. All of the treated teeth were vital at the end of the treatment. Seven molars (35% of the molars) required additional orthodontic treatment to optimize the final position of the second molar in the arch.

## 4. Discussion

The present work demonstrates the effectiveness of the above-proposed surgical-orthodontic technique for the retrieval of impacted mandibular second molars. The method differs from those proposed in the past and is characterized by innovative aspects, such as the use of a submerged device, high-strength titanium orthodontic coil springs, and the simultaneous extraction of ipsilateral third molars. Freeman et al. [14] used a spring vertically connected to a second molar buccal tube; this method is more invasive since it requires the placement of a separator between the first and second mandibular molars as well as the surgical removal of the mucosa or bone that covers the impacted molar.

Johnson et al. [15] described a complicated method that involved the surgical placement of separation wires; this technique allows only limited tooth movement and could cause periodontal problems. The same author also suggested cementing a partial crown on the exposed part of the second molar and uprighting it with the aid of springs. This seems a more viable and less invasive alternative, the main drawback of which (as mentioned by the author himself) lies in the need to expose part of the second molar to the oral cavity in order to ensure a precise impression and a reliable cementing of the partial crown.

Lee et al. [17] reported using one miniscrew with a collar diameter of 1.8 mm placed distally to the impacted tooth and a second miniscrew placed between the first and second premolars. The second molar is distalized by attaching a tube, which contains an open spiral coil, to the disto-buccal surface of the second molar. This technique is certainly more invasive than the one we propose because it requires attaching two miniscrews and replacing the coil spring every four weeks.

The method examined in the present paper originates from others described in the literature but aims to overcome their disadvantages. The starting point, similarly to the other previously proposed techniques, is the need to extract any ipsilateral third molars and simultaneously insert the miniscrew to optimize the surgical procedure and operating time. A relevant difference lies in the use of a 150–200 g coil spring instead of the 50 g one that Giancotti et al. used; the first one is obviously stronger. The safety and benefit that come from using a stronger spring seem to be confirmed by the fact that all second molars included in our sample remained vital during the medium-term follow-up [18].

A further original aspect of this method concerns the composition of the miniscrew; we opted for surgical steel instead of the commercially pure, grade 5 titanium used by other authors. Although previous studies did not report cases of titanium miniscrew osseointegration, it would be safer to use stainless steel miniscrews, given their need to be removed at the end of the orthodontic therapy.

The protocol used in the present work envisages the positioning of the miniscrew distal to the second molar, as a strategy to obtain a straighter and biomechanically more favorable traction force vector. Lee et al., Kravitz et al. and Derton et al. instead positioned their miniscrews mesially and completed molar uprighting with a sectional orthodontic wire [17,21,22]. For the purpose of controlling the traction, using a spring with a distal force vector seems to represent a more straightforward solution. Exerting the force directly on the tooth avoids any unwanted movement of the miniscrew anchoring unit, movement that can happen both as a result of technical errors in the passive attachment’s placement or as a weak attachment between the miniscrew and the tooth used as anchorage.

In our sample, the mean starting angle between the impacted tooth and the adjacent tooth was 55.7° (SD 22.6°). All impacted teeth reached an optimal final position, and additional orthodontic treatment was only required in a few cases. The starting angle in our patients was more than twice as wide as in the sample treated by Sonis and Ackermann, who reported treating 200 patients consecutively for mild-to-moderate crowding, using E-space, without any dental extractions [23]. Twenty-nine (14.5%) of their patients had at least one impacted second molar (24 had one, and 5 had two). Thirty-four (8.5%) out of a total of 400 teeth were classified as impacted. Prior to any treatment, the intermolar angle ranged from 19° to 33° (mean 24.6°, median 26°) for these impacted second molars (while it ranged from 24° to 33° (mean 14.5°, median 10.5°) for the sample as a whole). A statistically significant association emerged between pretreatment intermolar angle and mandibular second molar impaction (*p* < 0.001): an intermolar angle of 24° resulted in a positive predictive value of 1.0, and a negative predictive value of 0.92; an intermolar angle of 20° degrees coincided with the best sensitivity (94.12) and specificity (91.57) for second molar impaction. An extremely wide initial angle of 104.8° was corrected in one patient in our sample, demonstrating the efficacy of the proposed technique.

The main disadvantage of this technique lies in the size of the miniscrew, since it must be placed in a position where it may interfere with occlusion. However, this drawback seems to be almost negligible, since the head of the miniscrew is positioned just above the crest, where it represents a minimal encumbrance within the oral cavity.

No differences between males and females were observed in the outcomes we obtained. The only sex-related difference is represented by the higher frequency of second molar impaction in males. This findings are in contradiction with a systematic review conducted by Ramírez-Ossa et al., in which no differences between the two sexes were identified [24].

Among the encountered complications, the most common one is represented by mucosal hypertrophy around the orthodontic bracket, subsequent to the emergence of the second molar in the oral cavity. Many patients also had great difficulty in maintaining adequate oral hygiene at the intervention site.

The method presented in this paper proved to be minimally invasive, and once the third molar was extracted, all of the surgical procedures seemed to merge in a single fluid one. With the flap still open, the second molar is exposed, and the traction device is attached. The empty space in the alveolar process created distally to the second molar by the extraction of the third molar (or by means of a surgical procedure [20]) can promote and accelerate the second molar’s distalization movement.

The simple design of the device we described, and the short chair time required for its insertion, help minimize possible complications (compared to bulkier sectional devices). Exerting the traction force directly on the second molar avoids the risk of unwanted movements caused by any malpositioning of traction devices on adjacent teeth. The anchoring screw’s removal after the treatment is straightforward, with negligible risks and consequences for the patient.

## 5. Conclusions

Despite the limitations of a retrospective assessment of a case series, the present study demonstrates the efficacy of a novel technique for uprighting impacted second molars. This method represents a valid alternative to the surgical and orthodontic techniques already described in the literature. Given that the positioning of the miniscrew and of the orthodontic button on the second molar is imposed by needs of surgical anatomical access, the direction of the force vector of the coil spring is not ideal. The final optimal tridimensional position of the second molar could be achieved with a conventional fixed appliance. The use of this technique allowed us to correctly reposition all the impacted second molars that we treated, without incurring any serious complications.

## Figures and Tables

**Table 1:** Patients’ demographic details.

Patient Number.	Age at Time of Intervention	Sex	Tooth	Type of Impaction	Presence of Third Molar	Treatment Duration (months)	Side Effects	Additional Orthodontic Treatment	Initial Angle
1	19	M	3.7	intra-osseous	3.8	10	no	yes	53.4
2	16	M	3.7	mucous	3.8	10	no	no	29.4
3	18	F	3.7	mucous	3.8	9	no	yes	43.5
4	22	M	4.7	mucous	no	10	Ipertrofy of the mucosa	yes	36.8
5	18	M	4.7	intra-osseous	4.8	11	Poor hygiene	no	61.4
6	15	M	3.7	mucous	3.8	10	Ipertrofy of the mucosa	no	76.6
7	20	M	4.7	mucous	4.8	10	no	no	12.7
8	16	M	4.7	intra-osseous	4.8	14	no	no	64.6
9	13	M	3.7	mucous	3.8	4	no	no	46.3
10	20	M	4.7	mucous	4.8	11	no	no	61.4
11	16	F	4.7	mucous	4.8	10	no	yes	71.0
12	14	F	4.7	intra-osseous	4.8	9	no	no	67.8
13	18	F	3.7	mucous	3.8	10	no	no	104.8
14	23	F	4.7	intra-osseous	4.8	11	no	no	54.6
15	14	F	4.7	intra-osseous	4.8	9	no	no	73.6
16	21	M	4.7	intra-osseous	no	10	no	no	41.5
17	16	F	3.7	intra-osseous	3.8	9	no	no	13.3
18	21	F	4.7	intra-osseous	4.8	10	no	yes	49.3
19	14	M	3.7	intra-osseous	3.8	10	no	yes	75.7
	14	M	4.7	intra-osseous	4.8	10	no	yes	76.2

## Data Availability

Not applicable.

## References

[1] Neville B.W., Damm D.D., Allen C.M., Chi A.C. (2015). Oral and Maxillofacial Pathology.

[2] Soxman J.A., Wunsch P.B. (2019). Anomalies of Tooth Eruption. Clin. Dent. Rev..

[3] Grover P.S., Lorton L. (1985). The Incidence of Unerupted Permanent Teeth and Related Clinical Cases. Oral Surg. Oral Med. Oral Pathol..

[4] Varpio M., Wellfelt B. (1988). Disturbed Eruption of the Lower Second Molar: Clinical Appearance, Prevalence, and Etiology. ASDC J. Dent. Child..

[5] Cho S., Ki Y., Chu V., Chan J. (2008). Impaction of Permanent Mandibular Second Molars in Ethnic Chinese Schoolchildren. J. Can. Dent. Assoc..

[6] Bondemark L., Tsiopa J. (2007). Prevalence of Ectopic Eruption, Impaction, Retention and Agenesis of the Permanent Second Molar. Angle Orthod..

[7] Baccetti T. (2000). Tooth Anomalies Associated with Failure of Eruption of First and Second Permanent Molars. Am. J. Orthod. Dentofac. Orthop..

[8] Frank C.A. (2000). Treatment options for impacted teeth. J. Am. Dent. Assoc..

[9] Andreasen J.O., Kølsen Petersen J., Laskin D.M. (1997). Textbook and Color Atlas of Tooth Impactions: Diagnosis, Treatment, Prevention.

[10] Magnusson C., Kjellberg H. (2009). Impaction and Retention of Second Molars: Diagnosis, Treatment and Outcome. Angle Orthod..

[11] Eckhart J.E. (1998). Orthodontic Uprighting of Horizontally Impacted Mandibular Second Molars. J. Clin. Orthod..

[12] Shapira Y., Borell G., Nahlieli O., Kuftinec M.M. (1998). Uprighting Mesially Impacted Mandibular Permanent Second Molars. Angle Orthod..

[13] Suri L., Gagari E., Vastardis H. (2004). Delayed Tooth Eruption: Pathogenesis, Diagnosis, and Treatment. A Literature Review. Am. J. Orthod. Dentofac. Orthop..

[14] Freeman R.S. (1988). Mandibular Second Molar Problems. Am. J. Orthod. Dentofac. Orthop..

[15] Johnson J.V., Quirk G.P. (1987). Surgical Repositioning of Impacted Mandibular Second Molar Teeth. Am. J. Orthod. Dentofac. Orthop..

[16] Abate A., Cavagnetto D., Fama A., Matarese M., Bellincioni F., Assandri F. (2020). Efficacy of Operculectomy in the Treatment of 145 Cases with Unerupted Second Molars: A Retrospective Case–Control Study. Dent. J..

[17] Lee K.-J., Park Y.-C., Hwang W.-S., Seong E.-H. (2007). Uprighting Mandibular Second Molars with Direct Miniscrew Anchorage. J. Clin. Orthod..

[18] Giancotti A., Muzzi F., Santini F., Arcuri C. (2003). Miniscrew Treatment of Ectopic Mandibular Molars. J. Clin. Orthod..

[19] Bourzgui F. (2012). Orthodontics—Basic Aspects and Clinical Considerations.

[20] Finotti M., Del Torre M., Roberto M., Miotti F.A. (2009). La Distalisation Des Molaires Mandibulaires Peut-Elle Être Facilitée? Une Nouvelle Méthode Thérapeutique. Orthod. Française.

[21] Kravitz N.D., Yanosky M., Cope J.B., Silloway K., Favagehi M. (2016). Surgical Uprighting of Lower Second Molars. J. Clin. Orthod..

[22] Derton N., Perini A., Mutinelli S., Gracco A. (2012). Mandibular Molar Uprighting Using Mini-Implants: Different Approaches for Different Clinical Cases—Two Case Reports. Orthodontics.

[23] Sonis A., Ackerman M. (2011). E-Space Preservation: Is There a Relationship to Mandibular Second Molar Impaction?. Angle Orthod..

[24] Ramírez-Ossa D.M., Escobar-Correa N., Ramírez-Bustamante M.A., Agudelo-Suárez A.A. (2020). An Umbrella Review of the Effectiveness of Temporary Anchorage Devices and the Factors That Contribute to Their Success or Failure. J. Evid. Based Dent. Pract..

